# A novel and simple approach to the good process performance of methane recovery from lignocellulosic biomass alone

**DOI:** 10.1186/s13068-016-0530-1

**Published:** 2016-06-01

**Authors:** Yiqing Yao, Shulin Chen

**Affiliations:** Department of Biological Systems Engineering, Washington State University, Pullman, WA 99164 USA

**Keywords:** SS-AD, Simultaneous urea treatment, Rape straw, Soil, Methane yield

## Abstract

**Background:**

Solid-state anaerobic digestion (SS-AD) has been increasingly used for lignocellulosic biomass treatment. However, the separate reactor required for pretreatment prior digestion, poor treatment capacity, and process stability inhibit further development of the SS-AD. In this study, a novel method called SS-AD with simultaneous urea treatment and soil addition was proposed. The process performance of methane yield from rape straw was investigated by adopting the method.

**Results:**

The results show that the process performance of methane yield from rape straw using the method was better. The level of daily methane yield and the process stability were improved. The time required for reaching steady state was 6 days shorter than that of the common method (SS-AD and urea pretreatment), and the methane content in a stable-state level was 77.5–80.1 %. The total methane yield [409.6 L/kg volatile solids (VS)] was the maximal after using the method, which was 22.6 and 76.8 % higher than those of SS-AD with urea pretreatment and SS-AD with simultaneous urea treatment, respectively. In addition, the carbon dioxide content was reduced significantly. Degradation of feedstock was high; the highest reductions of VS, cellulose, and hemicellulose were 57.1, 61.4, and 65.8 %, respectively, which were in accordance with the maximal methane yield. SEM images also indicate that the biodegradation degree of rape straw in SS-AD was in line with methane yield.

**Conclusions:**

The process performance of SS-AD of lignocellulosic biomass (rape straw) with simultaneous urea treatment and soil addition was better. This simplified, low cost, and efficient method has good practicability, which can try to be used for other types of lignocellulosic biomass.

## Background

Global concerns over sustainability of petroleum supply and climate change have inspired world wide research and development of alternative energy source for fuel and energy production. Renewable energy resource represent about 14 % of primary energy consumption in the world, such as wind, wave, hydro, solar, biomass, and geothermal. As the matter for bioenergy yield, biomass occupies approximately about 10 % of the total energy and mainly contributes to renewable energy [1]. Lignocellulose, such as agricultural and forestry wastes, energy crops, and municipal solid waste, is the most abundant organic material and is widely available on the earth, which can be converted to various forms of fuel and energy; therefore, it is a promising raw material for bioenergy production [2].

Solid-state anaerobic digestion (SS-AD), with high total solid (TS) content of 20–55 %, has been successfully implemented in Europe for treating municipal solid waste since the early 1990s [3]. In recent years, various types of lignocellulosic biomass have been well handled by SS-AD for biogas yield. The problems encountered in liquid AD, such as floating and stratification of solids, can be avoided in SS-AD [4]. SS-AD also has other advantages over liquid AD, for example, less energy input, higher TS content, less water resource demand, and smaller working volume. However, the disadvantages of SS-AD are longer retention time, large amounts of inoculum (up to 50 %), and low efficiency of substrate utilization, when lignocellulosic biomass is used [5, 6]. However, in view of practical application, suitable nitrogen rich materials used for co-digestion with lignocellulosic biomass in a certain region are often difficult to collect [7]. That is to say, SS-AD of lignocellulosic biomass alone is necessary. Although SS-AD with lignocellulosic biomass as sole feedstock is necessary, very few attempts have been made. The main reason is the complex three-dimensional structures that was formed of polysaccharide (cellulose and hemicellulose) and lignin, and creates recalcitrant to fermentative microorganism, and then, the inhibition of hydrolysis of AD [8, 9]. Alkaline pretreatment is one of the leading methods, because it has many advantages, for example, alkaline can solubilize lignin, neutralize various acidic products, and prevent drop of pH during subsequent acidification process [10, 11]. It has been reported that a 37.0 % increase in biogas was achieved by Zhu et al. [12], with 5.0 % NaOH-treated corn straw at 53.0 % moisture content for 1 day at ambient temperature. Recently, it has been shown that a 113.8 % higher of methane yield than non-alkaline treated poplar processing residues was obtained at conditions of 35 g/L and 5.0 % NaOH loading [13]. However, studies of SS-AD of lignocellulosic biomass with simultaneous treatment are few. To simplify the operation by eliminating a separate reactor for pretreatment and reduce the capital cost, simultaneous treatment is necessary and significant for the purpose of practical application. Disappointingly, to date, the successful results have not been reported. Zhu et al. [12] tested SS-AD of corn straw with simultaneous NaOH treatment at conditions of 18 of C/N ratio and 5.0 % NaOH loading, but the improvement in biogas yield (only 9.0 % of increase) was not obvious compared with that of untreated corn straw. Later, Liew et al. [14] tried to investigate SS-AD of fallen leaves with simultaneous alkaline treatment by adjusting S/I ratio, the result was good, but the amount of inoculum in reactor was large, which reduced the effective working volume in a certain working volume.

Up to now, problems present in SS-AD of lignocellulosic biomass with simultaneous treatment have not been satisfactorily solved. As a type of familiar material, the properties of soil may be beneficial for solving the problems. Multiple elements required by microorganisms as nutrients are contained in soil, such as carbon, nitrogen, phosphorus, sodium, potassium, calcium, and magnesium [15]. Importantly, soil has buffering capacity, which can maintain a stable status of systems by neutralizing acids and bases [15]. In other words, soil has the potential of enhancing activity of microorganisms in bioreactor by providing multiple elements, and maintaining a stable system of bioreactor. In addition, soil is conveniently available, the amount of which is abundant, and can be handily collected without paying. In this study, urea was used for simultaneous treating lignocellulosic biomass (rape straw). At the same time, urea can be used as nitrogen source in SS-AD of rape straw with simultaneous treatment. If this hypothesize is feasible, the attraction of this novel method will be good for both theoretical research and practical application: (1) a separate reactor for pretreatment can be eliminated, so the operation of AD can be simplified; (2) problem about process stability can be overcome, which is common for SS-AD of lignocellulosic biomass; (3) water resource can be economized; (4) less waste water emission; and (5) lignocellulosic biomass can be efficiently utilized for energy recovery (methane yield).

## Results and discussion

### Solid-state anaerobic digestion with simultaneous urea treatment and soil addition

#### Daily methane yield

**Fig. 1:**
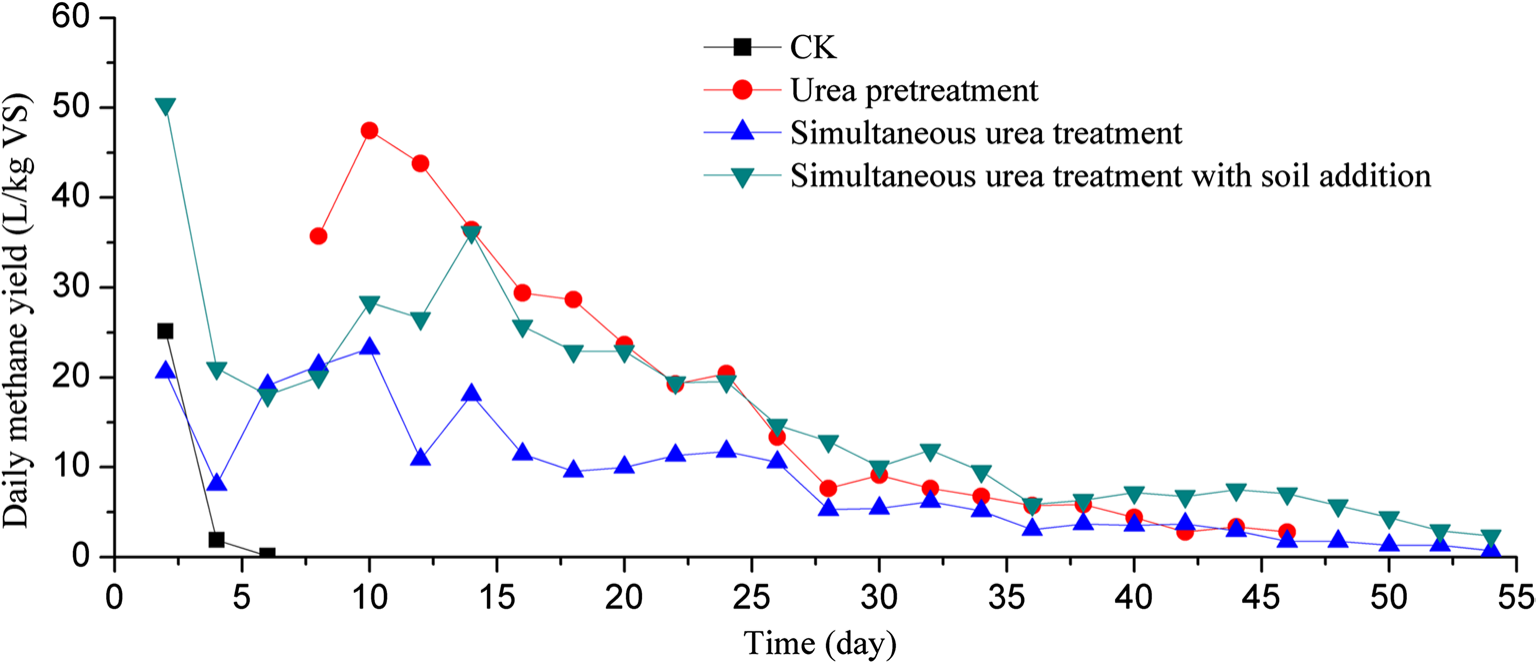
Daily methane yield of untreated and different treated rape straws. *CK* Sample with no urea treatment and no soil addition; *Urea pretreatment* sample with urea treatment prior to AD and no soil addition; *Simultaneous urea treatment* urea treatment along with AD and no soil addition; *Simultaneous urea treatment* urea treatment along with AD and soil addition

The trends of daily methane yield of experiments were similar, but with some differences (Fig. 1). The daily methane yield of no urea treatment (CK) appeared continuous decrease and ceased on day 6. This phenomenon was also occurred for other experiments. This may be the accumulation of volatile fatty acids (VFAs). First, different from the experiments with urea addition, the nutrient was not balance for CK due to no urea addition; therefore, the C/N ratio of CK was 40.1 and was much higher than the appropriate range. According to Bardiya et al.’s [16] report, the suitable C/N ratio was 20–30:1. Many studies shown that if the C/N ratio is too high, accumulation of VFAs occurs, this leads to the inhibition of AD. For example, Molinuevo-Salces et al. [17] studied anaerobic co-digestion of swine manure and vegetable wastes; they found the strong inhibition phenomenon due to the highest concentration of VFAs, when the treatment was characterized by high vegetable wastes content. Liew et al. [18] investigated the effect of S/I (substrate-to-inoculum) ratio on AD and found the SS-AD process failure, when the fallen leaves were increased to high level (S/I = 8.2), this is because of the high total VFA in the digestate. We studied the anaerobic co-digestion of Solidago Canadensis L. biomass (SC) and cattle slurry (CM), the result shows that the failure of AD is resulted by the high C/N ratio (SC: CM of 3:1) [19]. Second, the high VS content of 91.2 % (Table 1) is beneficial for biodegradation of rape straw and methane yield. Third, the pH of rape straw (pH = 6.5) is low, which is unfavorable for SS-AD. As a result, these factors can result in the accumulation of VFAs, and then, the low pH (pH = 5.5) of the system for CK. The low pH was not in the appropriate range (6.8–7.5) for a stable AD [20]. In a bioreactor, the microbial consortium consists of fermentative bacteria, acetogenic bacteria, and methanogenic archaea. Among these microorganisms, the grow rate of methanogenic archaea is lower than that of others, methanogenic archaea are, therefore, sensitive to the changes of environmental conditions [21]. Similar phenomenon also appeared for the experiment with urea pretreatment, there was no methane yield in the initial period, but daily methane yield appeared from day 6, which was not like that of CK. This can be attributed to the readily biodegradable organic matter from the urea pretreated rape straw, the amount of acidic products was also large due to low pH (pH = 6.15). The large amount of intermediate products from the urea pretreated rape straw inhibited the activity of methanogenic archaea; then, the destruction of microbial communities in bioreactor. As a result, the system of bioreactor could not work well. After a long period of adaption and breeding of methanogenic archaea, most of the available substrate was steadily consumed during the methane fermentation process, the daily methane yield increased accordingly, but early ceased on day 46. However, the daily methane yield of CK ceased from day 6 until to the end of SS-AD, this is because of no urea addition. Urea could provide balance nutrients or suitable C/N ratio for SS-AD of lignocellulosic biomass; the suitable C/N ratio could maintain the activity of methanogenic archaea, and then the satisfactory evolvement of SS-AD process. For experiments with simultaneous urea treatment, the daily methane yields were continuous throughout the process of SS-AD, which were not like CK and experiment with urea pretreatment. This can be attributed to the slowly release of intermediate products during the process of simultaneous urea treatment; therefore, the amount of the products were not large and could be efficiently utilized by microbes. It can be seen that level of daily methane yield for SS-AD with simultaneous urea treatment and soil addition was higher than that of SS-AD with simultaneous urea treatment and no soil addition. The fluctuation of daily methane yield for the experiment with simultaneous urea treatment without soil addition was obvious and huge along the whole process. This phenomenon is common for SS-AD of lignocellulosic biomass with simultaneous treatment. Zhu et al. [12] studied SS-AD of corn straw with simultaneous alkaline treatment and found the level of daily biogas yield was lower than that of the untreated one, with 5.0 % NaOH loading. Liew et al. [14] investigated the effect of NaOH loading on daily methane yield at different S/I ratios and found that the huge fluctuation of daily methane yield appeared for all simultaneous alkaline treatments at both 4.1 and 6.2 of S/I ratios, and even the cease of daily methane yield occurred for simultaneous 2.0 % NaOH treatment at 6.2 of S/I ratio. From the above, in addition to the similar trends of daily methane yields, all the treatments had clear peaks during fermentation. For the initial stage of fermentation, the daily methane yield increased rapidly followed by a temporary rapid decrease except for the urea pretreatment, then a gradual increase. This is because of the readily biodegradable organic matter of feedstock available for anaerobic microbe initially [12]. When the available matter was nearly used out, decrease of the daily methane yield appeared accordingly [22]. In addition, at the initial stage of AD, the VFAs concentrations can increase to high levels; this phenomenon is typical for the start-up stage of AD, because of the imbalances among hydrolytic, fermentative, acetogenic, and methanogenic functions during this period [14, 23]. When VFAs were steadily consumed, methane production increased accordingly. This phenomenon was verified by many studies. Molinuevo-Salces et al. [17] and Yao et al. [19] found the accumulation of VFAs and the decrease of daily methane yield at the start-up stage followed by low level of VFAs until to the end of AD. Along with the steadily consumed of VFAs and further solubilization of feedstock, the increase of daily methane yield after a period of acclimation and breeding of hydrolytic bacterium appeared. Then, the daily methane yield for the three treatment maintained decrease until to the end along with the continued consumption of feedstock.

**Table 1:** Characteristics of rape straw, inoculum and soil

Parameter	Rape straw	Inoculum	Soil
Total solid (%)	96.0 ± 0.0	5.5 ± 0.1	83.3 ± 0.0
Volatile solid (%)	91.2 ± 0.6	68.8 ± 0.9	3.8 ± 0.0
Total carbon (%)	44.5 ± 0.1	35.9 ± 0.1	2.6 ± 0.1
Total nitrogen (%)	0.8 ± 0.0	1.8 ± 0.0	0.06 ± 0.0
H (%)	6.0 ± 0.3	4.7 ± 0.2	0.4 ± 0.0
pH value	6.5 ± 0.0	7.8 ± 0.1	6.9 ± 0.0
Cellulose (%)	49.3 ± 0.0	33.0 ± 0.0	ND
Hemicellulose (%)	18.9 ± 0.0	25.6 ± 0.2	ND
Lignin (%)	21.5 ± 0.0	ND	ND

*ND* not determined

#### Methane content

**Fig. 2:**
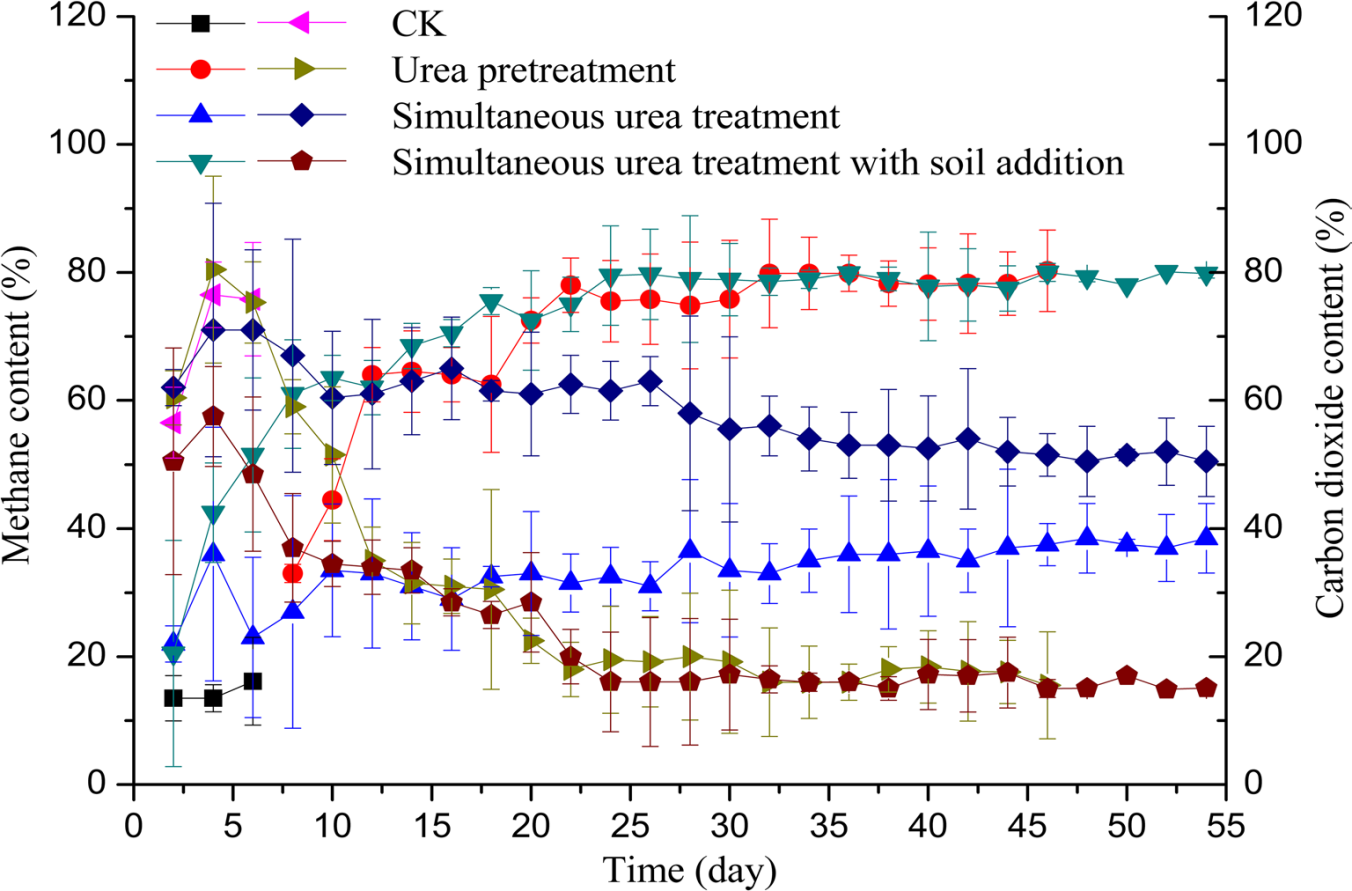
Methane content and carbon dioxide content of untreated and different treated rape straws. Symbols of the *left column* belong to methane content; symbols of the *right column* belong to carbon dioxide content

Methane contents of experiments were obviously different from each other (Fig. 2). For the SS-AD of rape straw with urea pretreatment, methane content increased until to day 12, and then decreased slightly following by rapid increase. This may be the inhibition of SS-AD due to the amount of acidic products yield from the urea pretreatment process. The methane content maintained stable from day 22 on and was 75.5–80.3 %. Obviously, the methane content of SS-AD with simultaneous urea treatment was much lower than that of SS-AD with simultaneous urea treatment and soil addition. The methane content for simultaneous urea treatment without soil addition maintained a low level throughout the process of SS-AD, and was lower than 50.0 %, the range of methane content was 23.0–30.5 %. Superior to 50.0 % of methane content indicates that the SS-AD process was in a stable state [12]. For the microorganisms of fermentative bacteria, acetogenic bacteria, and methanogenic archaea in a bioreactor, the grow rate of methanogenic archaea is lower than others; therefore, the methanogenic archaea are sensitive to the changes of environmental conditions [21]. Therefore, the lowest methane content (23.0–30.5 %) means the system of bioreactor worked unhealthy and the activity of methanogenic archaea was inhibited. This result underlines that the whole process of SS-AD with simultaneous urea treatment and no soil addition was not stable. Contrarily, for the experiment with soil addition, methane content reached steady state on day 6, and the fluctuation of methane content appeared between day 10 and day 12. This was in accordance with the corresponding daily methane yield. After that, the methane content increased continuously until to day 24 and maintained a stable level and was 77.5–80.1 %. In addition to the experiment with soil addition, methane content for the SS-AD with simultaneous urea treatment and no soil addition also reached steady state on day 12, but the time used for reaching the steady state was 6 days longer than that of the experiment with simultaneous urea treatment and soil addition.

#### Carbon dioxide content

The carbon dioxide contents were shown in Fig. 2. For all the treatments, carbon dioxide contents increased initially and then decreased until to stable levels. Especially, for the simultaneous urea treatment with soil addition, the level of carbon dioxide content at the initial period of AD was the lowest; this is in accordance with the quick speed-up stage in AD. However, for the simultaneous urea treatment without soil addition, the level of carbon dioxide content was the highest nearly during the whole process of AD. That is to say, the higher levels of CH_4_ contents correspond to the lower levels of carbon dioxide contents for all the treatments. The carbon dioxide content of simultaneous urea treatment with soil addition was the lowest during the whole process of AD. The reasonable consideration is that the activity of methanogens was activated by soil addition, then much more carbon dioxide be converted to CH_4_. Therefore, the carbon dioxide content was much less. Chuang et al. [24] and Zhen et al. [25] analyzed the carbon dioxide emission reduction efficiency detailedly and concluded that the energy produced from biomass could mitigate the carbon dioxide emission reduction from the non-renewable (coal and fuel oil), therefore, the further reduced carbon dioxide emission in this study will be significant.

#### Total methane yield

**Fig. 3:**
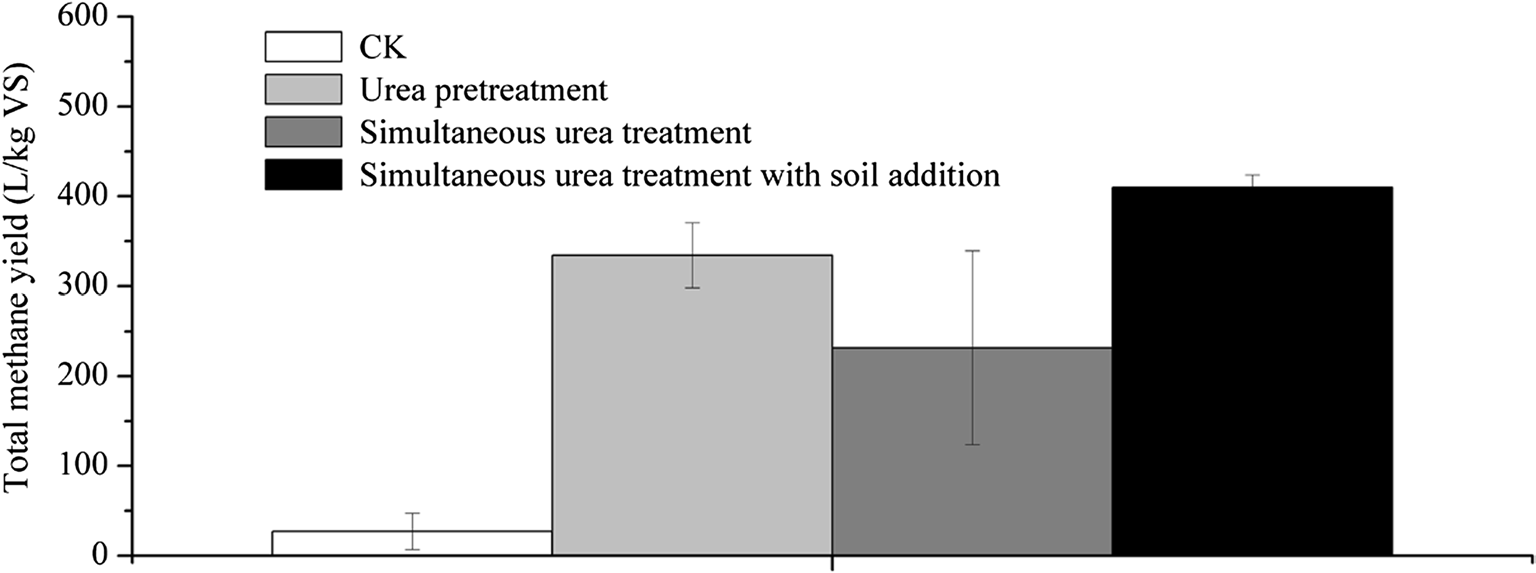
Total methane yield of untreated and different treated rape straws

In view of the total methane yield, urea addition could maintain the operation of SS-AD with both urea pretreatment and simultaneous urea treatment (Fig. 3). For the experiments of urea pretreatment, simultaneous urea treatment, and simultaneous urea treatment with soil addition, the total methane yields were 334.2, 231.7, and 409.6 L/kg VS, respectively. Therefore, the maximal methane yield was obtained for SS-AD with simultaneous urea treatment and soil addition, which were 22.6 and 76.8 % higher than those of SS-AD with urea pretreatment and SS-AD with simultaneous urea treatment, respectively. The minimal methane yield for SS-AD with simultaneous urea treatment and no soil addition was in accordance with the corresponding daily methane yield and methane content, both of the daily yield and the methane content were in the lowest level among all the experiments. Zhu et al. [12] studied the potential of biogas yield from SS-AD of fallen leaves with simultaneous alkaline treatment, the result shown that the maximal biogas was obtained at 2.5 % NaOH loading and was 300.7 L/kg VS, the total methane yield was certainly lower than 300.7 L/kg VS. Liew et al. [14] investigated the potential of methane yield from corn straw with the same method (SS-AD with simultaneous alkaline treatment); the maximal methane yield was only 82.0 L/kg VS. Thus, it can be seen that the maximal methane yield in this study (409.6 L/kg VS) was much higher than the previous results. As a result, soil could significantly improve the methane yield for SS-AD of rape straw with simultaneous urea treatment.

Based on the above, the benefits of soil for SS-AD of rape straw with simultaneous urea treatment were obvious. In soil, multiple elements required by microorganisms as nutrition are available. The buffering capacity of soil is similarly important, when acidity increase, more H^+^ ions are attached to the colloids; at the same time, other cations are pushed away from the colloids. The strength of absorption of different cations is generally in the following order [26]:

$${\text{Al}}^{ 3+ } > {\text{H}}^{ + } > {\text{Ca}}^{ + } > {\text{Mg}}^{ 2+ } > {\text{K}}^{ + } = {\text{NH}}_{ 4}^{ + } > {\text{Na}}^{ + } .$$

In summary, effects of simultaneous urea treatment with soil addition are good according to the analysis of the methane production performance: soil addition was useful for enhancing the process stability and improving the level of daily methane yield of SS-AD of lignocellulosic biomass with simultaneous urea treatment; soil addition efficiently improved the methane content and reduced the time required for reaching steady state; the carbon dioxide content emission was also reduced; importantly, the total methane yield was enhanced.

#### Degradation of feedstock

It can be seen that the feedstock degradation was in line with the total methane yield (Table 2); higher feedstock degradation was associated with higher total methane yield. However, TS reductions were different, which were not in line with the total methane yields. The lowest TS reduction was associated with the maximal methane yield. This is because of soil addition, which contains low VS content (3.8 %). In this study, the highest reductions of VS, cellulose, and hemicellulose were 57.1, 61.4, and 65.8 %, respectively, which was higher compared with previous results. Liew et al. investigated SS-AD of fallen leaves with simultaneous alkaline treatment and found the highest cellulose and hemicellulose degradations were 36.0 and 34.9 %, respectively [14], which were 41.4 and 47.0 % lower compared with the corresponding values in this study. This result indicates that soil could significantly improve the efficiency of substrate utilization for methane yield.

**Table 2:** TS, VS, cellulose and hemicellulose degradations (%) of feedstock after anaerobic digestion

Parameter	Untreated	Urea pretreatment	Simultaneous urea treatment	Simultaneous urea treatment and soil addition
TS	–	43.5 ± 2.1	30.1 ± 0.2	20.4 ± 0.5
VS	–	49.4 ± 1.1	35.3 ± 1.1	57.1 ± 0.0
Cellulose	–	48.6 ± 1.5	37.0 ± 0.7	61.4 ± 0.1
Hemicellulose	–	55.0 ± 0.2	40.6 ± 1.2	65.8 ± 0.3

#### Scanning electron microscopy (SEM)

**Fig. 4:**
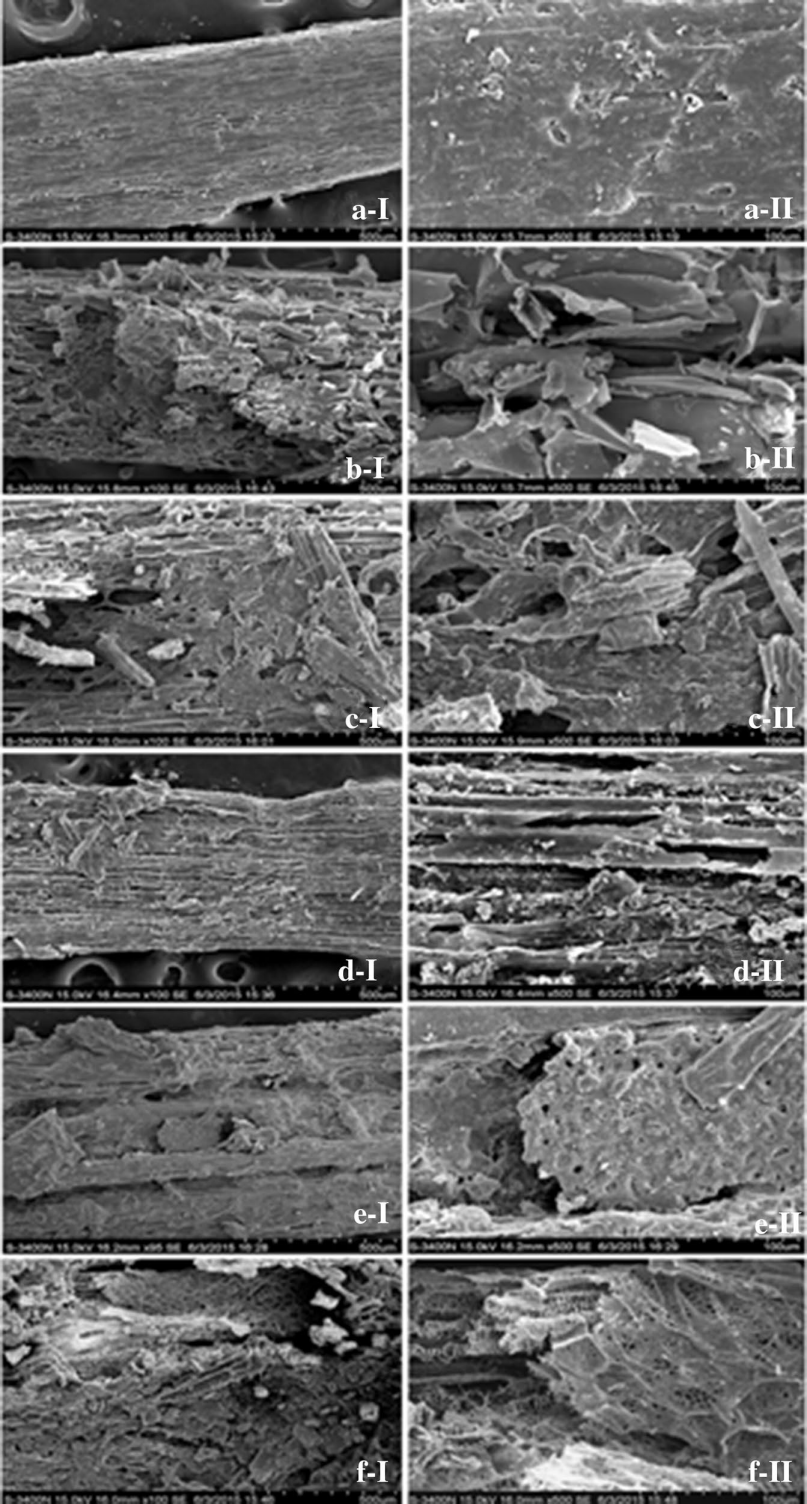
SEM photos of rape straw. **a** Raw straw; **b** urea pretreatment; **c** fermented without treatment; **d** fermented with simultaneous urea treatment; **e** fermented with urea pretreatment; **f** fermented with simultaneous urea treatment and soil addition. *I* 100×; *II* 500×

The physical structure of unfermented and fermented rape straw samples was studied by SEM images (Fig. 4). The texture of raw sample exhibited rigid and highly ordered fibrils, and the surface was covered with a thin film, maybe the film is wax layer, which is commonly found in herbaceous biomass (Fig. 4a) [27]. After treatment by urea, the thin film (wax layer) on the surface disappeared completely, the texture was unordered and fragmented, and some holes appeared on the surface of solids. This indicates that the structure of sample was disrupted by urea treatment to a great extent. The changes were beneficial for the efficient biodegradability and the utilization of rape straw in later SS-AD. As shown in Fig. 4e, after SS-AD, the available organic matter for microbes was completely utilized; the rest of structure appeared rigid, which could not be degraded by microbes in SS-AD. However, when raw sample was used as substrate of SS-AD for methane yield, the texture of rape straw after SS-AD appeared no great changes. Although the wax layer was completely disappeared, many short fibers were still on the surface of digested solids and were not degraded efficiently in SS-AD (Fig. 4c). Similarly, texture of digested solids for SS-AD with simultaneous urea treatment still exhibited rigid and compact, and appeared ordered generally (Fig. 4d). Unlike the above, for SS-AD with simultaneous urea treatment and soil addition (Fig. 4f), the sample was more thoroughly exhausted by microbes, clear network structures appeared softer and account for most of the digested rape straw. This great change was associated with the maximal methane yield. These results show that soil addition is beneficial for the efficient biodegradability of rape straw and then the efficient methane yield.

## Conclusion

The benefits of the novel method (SS-AD with simultaneous urea treatment and soil addition) for methane yield from lignocellulosic biomass (rape straw) were obvious: for the economic benefits, (1) the treatment stage and the SS-AD can be conducted in single reactor, so the operation was simplified and the capital cost was reduced; (2) the steady state of daily methane yield was enhanced; (3) the time required for reaching stable state was reduced or the start-up stage was hastened; (4) the methane content and the total methane yield were improved; (5) the carbon dioxide content was reduced significantly, which leads to the reduction of carbon content and the reduction of costs for biogas purification; (6) the water resource was economized due to no large amount of water required for SS-AD; and (7) the cost for collecting soil is low, for the environmental benefits, on one hand, there is no need to wash the lignocellulosic materials, which is usually required for the stage of pretreatment; so, there is no emission of waste water; on the other hand, there is no large amount of waste water produced after SS-AD due to the high substrate concentration. Therefore, the strategy has potential feasibility for full-scale application. It is a cost-effective and environmental friendly technique, and can realize maximal resource recovery and methane yield in the field of bioenergy recovery from lignocellulosic biomass. Significantly, it opens a door to efficiently and low-costly utilize lignocellulosic biomass alone by SS-AD.

## Methods

### Feedstock and inoculum

The rape straw was collected from Yuzhong County of Lanzhou City, Gansu province, China. The rape straw was cut and grounded into 6–12 mm particles by a hammer mill (RT-34, BeijingWeiBoChuang, China). The resultant rape straw was stored at −20 °C prior to use. Effluent from a biogas plant digesting manure in Linxia city, Gansu province, China, was used as inoculum in this study. Soil samples were collected from the campus of Lanzhou University and were ground into powder. The characteristics of the corn straw, inoculum, and soil are shown in Table 1.

### Solid-state anaerobic digestion with simultaneous urea treatment and soil addition

The rape straw was digested in batch anaerobic digesters at laboratory scale. The volume of each digester was 2 L. Ground rape straw was mixed thoroughly with an appropriate amount of inoculum effluent and urea pellets to achieve 4.1 of S/I ratio (based on VS) [14]. The TS of rape straw for each digester was 30 g. Urea was added into each digester to obtain a C/N ratio of 25:1 [28]. The amount of urea added to each digester was 0.55 g. Soil was added into one digester to obtain 2.5:1 of rape straw/soil (based on dry matter) [7]. For the simultaneous urea treatment, urea was predissolved in effluent and then added to reactors. Reactors without soil addition for simultaneous urea treatment, urea pretreatment, and no urea treatment (CK) were also conducted in parallel. For the urea pretreatment, the required amount of urea was used, the moisture content was 80 %, and the prepared samples were kept at ambient temperature (20 ± 1 °C). After 4-day pretreatment, the pH reached to near 7.0, which means that there is no need to adjust the pH before AD; so, the time used for pretreatment is 4 days. The headspace of the digesters was flushed with nitrogen gas for about 5 min to obtain anaerobic conditions before incubation; then the digesters were capped tightly with rubber stoppers. The prepared digesters were operated at 37 °C (mesophilic temperature) without shaking, which was the optimal temperature for AD [29]. The digestion experiment for each condition was triplicated.

### Analytical methods

#### Chemical composition analyses

TS and VS were determined according to the APHA standard methods (1998) [30]. Total carbon (TC), total nitrogen (TN), and total hydrogen (TH) were measured by an elemental analyzer (varioEL cube, Elementar Analysensysteme GmbH). The samples were prepared by suspending 5-g wet digestate into 50-ml distilled water; then, the pH determination by pH meter (PB-21, Sartorius, Germany) [12]. The contents of cellulose, hemicellulose, and lignin were determined according to the procedure of Van Soest et al. [31].

#### Biogas analyses

Method of water displacement was used for recording volume of biogas every 2 days; the total biogas volume was calculated after anaerobic co-digestion. The CH_4_ % was analyzed using a gas chromatograph (GC) (Agilent Technologies, 7890A, Wilmington, DE, USA) equipped with a thermal conductivity detector (TCD) and a 25 m × 530 × 20 mm chromatographic column. Hydrogen (35 ml/min) was used as the carrier gas. The temperatures of injector port and detector were 75 and 150 °C, respectively. The standard gas (YQD-09, QingdaoHuaQing Co., Shandong, China) is composed of 30.1 % N_2_, 39.9 % CH_4_, and 30.0 % CO_2_.

#### Scanning electron microscopy (SEM)

The microscope photos of samples were taken by a Model S-3400 N scanning electron microscopy (Hitachi, Japan) after the samples sputter-coated with a thin layer of gold.

### Statistical analysis

The software spss 19.0 was used to determine the standard deviations and whether the observed differences between two or more groups of experimental results were significant. Differences were compared with p value of 0.05.

## Abbreviations

AD: anaerobic digestion
SS-AD: solid-state anaerobic digestion
S/I: substrate-to-inoculum
CK: no urea treatment
VFAs: volatile fatty acids
SEM: scanning electron microscopy
TS: total solids
VFAs: volatile fatty acids
VS: volatile solids
